# Intestinal type adenocarcinoma of the endometrium with signet ring cells, a rare aggressive variant

**DOI:** 10.1016/j.gore.2022.101046

**Published:** 2022-07-21

**Authors:** Kieran Seay, Bethany Bustamante, Alexander Truskinovsky, Andrew Menzin, Gary L. Goldberg

**Affiliations:** a Division of Gynecologic Oncology, Department of Obstetrics and Gynecology, Northwell Health, Zucker School of Medicine at Hofstra/Northwell, Long Island, NY, United States; b Pathology and Laboratory Medicine, Northwell Health, Zucker School of Medicine at Hofstra/Northwell, Long Island, NY, United States

**Keywords:** Endometrium, Intestinal differentiation, Signet-rings morphology, E-cadherin

## Abstract

**Objective:**

Intestinal type mucinous adenocarcinoma (iMACE) is a rare and unusual variant of mucinous carcinoma of the endometrium that can show focal features of poorly differentiated adenocarcinomas of gastric, pancreatic or intestinal origin by producing signet ring cells. To date, only two reported cases of signet ring cells as a morphological feature of iMACE have been reported. Alterations in E-cadherin expression have been linked to increased metastatic potential, tumor dedifferentiation, and deep myometrial invasion in endometrial carcinomas. The presence or absence of E-cadherin in iMACE with signet-ring cells has not been studied. Thus, we sought to analyze E-cadherin expression in this aggressive variant of endometrial carcinoma.

**Cases:**

Diagnosis of iMACE with signet ring cells was rendered with the aid of immunohistochemical staining and histological analysis. Average age of diagnosis was 72 with the presenting complaint of postmenopausal bleeding in all three women. Focal loss or weakly positive E-cadherin expression was seen in areas of signet-rings cell morphology in all three cases.

**Conclusion:**

This case report adds to the body of literature that demonstrates the aggressive nature of intestinal differentiation in the endometrium. In addition, this report suggests that the presence of signet-rings cells and loss of E-cadherin expression may render a poorer prognosis as seen in other parts of the body.

## Highlights

• Intestinal type mucinous adenocarcinoma is a rare variant of mucinous carcinoma of the endometrium.
• Intestinal differentiation in the endometrium can manifest with a wide spectrum of morphologies including signet-ring cells.
• E-cadherin expression is downregulation in the signet-ring cell component in all three cases.
• Cases described provides further evidence of the aggressive nature of intestinal type mucinous adenocarcinoma.

## 1 Introduction

Endometrial cancer is the most common gynecological malignancy in the United States. Over 63,000 new cases are reported annually and 1–9% are reported to be mucinous adenocarcinoma of the endometrium (MACE). According to the 2014 World Health Organization (WHO), mucinous adenocarcinoma is defined as an endometrial carcinoma with >50% cells showing mucinous differentiation. In addition, the WHO suggests that mucinous adenocarcinoma is an independent category of endometrial cancers and not a subcategory of endometrioid endometrial carcinoma (Ardighieri et al., 2020).

Intestinal type mucinous adenocarcinoma (iMACE) is a rare and unusual variant of mucinous carcinoma that was first reported by Berger in 1984 (Berger, 1984). Recently, an exhaustive systematic review of the literature performed by Ardighieri reported a total of 9 cases (Ardighieri et al., 2020, Berger, 1984, Trippel et al., 2017, Rubio et al., 2016, Nieuwenhuizen, 2007, Buell-Gutbrod, 2013, Mogor et al., 2019, Zheng et al., 1995). Of these cases, only Berger reported the presence of signet-ring cells as a morphological feature of intestinal type mucous adenocarcinoma. Berger reported no evidence of disease on follow up of their cases (Berger, 1984). To our knowledge, these are the only cases of intestinal type mucinous adenocarcinoma with signet ring morphology and thus prognosis is difficult to determine. In other parts of the body signet-ring cell carcinoma (SRCC) is usually associated with a high-grade tumor and has a poor prognosis (Boyd et al., 2010, Wang et al., 2016). Interestingly, loss of E-cadherin expression in colorectal SRCC has been shown to be a independent prognostic factor of patients’ overall survival. We describe in this case report the loss of E-cadherin expression and the presence of signet-ring cells in three cases of intestinal type mucinous adenocarcinoma.

## 2 Case presentation

**Fig. 1:**
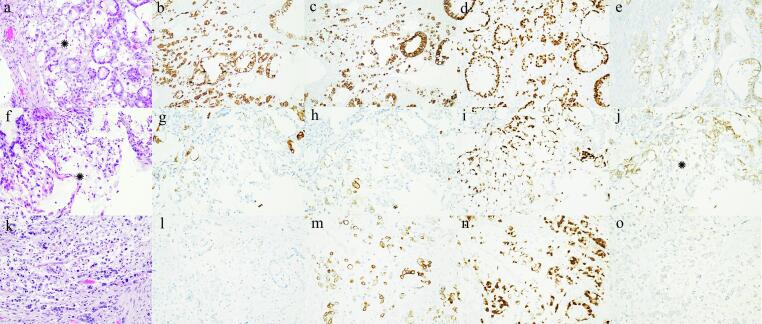
(a–e) Case A. (a) Hematoxylin and eosin-stained section shows focal signet ring cell carcinoma (asterisk) surrounded by moderately differentiated mucinous adenocarcinoma, morphologically compatible with the intestinal type (original magnification x200). Both components of the adenocarcinoma are positive for cytokeratin 7 (b) and cytokeratin 20 (c) (both original magnifications x100). (d) Both components of the adenocarcinoma are positive for CDX2 (original magnification x200). (e) The signet ring cell component of the carcinoma is focally, very weakly positive for E-cadherin (original magnification x200). (f–j) Case B. (f) Hematoxylin and eosin-stained section shows focal signet ring cell carcinoma (asterisk). The signet ring cell component is focally, weakly positive for cytokeratin 7 (g) and cytokeratin 20 (h), and diffusely positive for CDX2 (i). (j) The signet ring cell carcinoma is focally, very weakly positive for E-cadherin (asterisk). All original magnifications x200. (k–o) Case C. (k) Hematoxylin and eosin-stained section shows a prominent signet ring cell component of the adenocarcinoma, more abundant than in the previous two cases, showing high-grade nuclear atypia. The carcinoma is negative for cytokeratin 7 (l) and positive for cytokeratin 20 (m), showing a colorectal or appendiceal immunophenotype. (n) The tumor is positive for CDX2. (o) The signet ring cell carcinoma is completely negative for E-cadherin. All original magnifications x200. In all three cases, the signet ring cell component of the adenocarcinoma was negative for PAX8 and estrogen receptors (not shown).

Case A An 80-year-old para 9, with no past gynecologic care, presented to the emergency department with the complaint of one week of post-menopausal bleeding. A pelvic ultrasound was performed which showed a markedly distended and abnormal endometrium measuring 3.3 cm, and an enlarged right ovary measuring 4 cm without discrete mass. She was referred to a gynecologist who performed an endometrial biopsy which showed FIGO grade 2 endometrioid adenocarcinoma with focal mucinous features. She was brought to the operating room by a gynecologic oncologist who performed total abdominal hysterectomy, bilateral salpingo-oophorectomy, right pelvic lymph node excision, omentectomy and appendectomy. Findings at the time of surgery included a ten-week gestational size uterus with irregular contour, enlarged right ovary grossly suspicious for tumor involvement, enlarged right common iliac lymph node and grossly normal appendix and omentum. Frozen section of the right ovary revealed adenocarcinoma with possible signet-ring cells. Due to this histologic finding, an appendectomy was performed. Excision of the enlarged right common iliac lymph node was performed and node was found to be positive for metastatic adenocarcinoma on frozen section. There were no other enlarged lymph nodes and systematic lymphadenectomy was deferred. The patient recovered well post-operatively. Final pathology demonstrated gland forming architecture with focal intestinal type mucinous glandular differentiation and signet-ring cells. The tumor was deeply invasive through the myometrium, involved cervical stroma, bilateral ovaries, right pelvic lymph node, and omentum. Peritoneal cytology was negative. The signet ring cell component of the carcinoma was positive for both cytokeratin 7 and cytokeratin 20, positive for CDX2, negative for PAX8 and estrogen receptors, and focally, very weakly positive for E-cadherin (Fig. 1). The staining for the DNA mismatch repair (MMR) proteins was intact. A upper and lower gastrointestinal workup was completed which included endoscopy, colonoscopy, and relevant imaging with results negative for a gastrointestinal primary malignancy. The patient declined chemotherapy treatment and she died eleven months post-surgery from diffuse metastatic disease to the lung, liver, bones, and brain.

Case B A 65-year-old para 0, who was compliant with routine well-woman gynecologic care, presented to her gynecologist with the complaint of several months of post-menopausal bleeding. She had a pertinent gynecologic history of cervical dysplasia that was treated with cryotherapy in the distant past, with normal Pap smears subsequent. A pelvic ultrasound showed an endometrial thickness of 12 mm, and endometrial biopsy was interpreted as adenocarcinoma, mucinous type with focal areas appearing endometrioid, but the primary site of origin was indeterminable. Tumor markers CA-125 and CEA were checked and within normal limits, 6 and 0.9 respectively. The patient underwent CT imaging of the abdomen and pelvis, which revealed a 1.9 cm right lateral uterine wall mass. Pre-operative endoscopy and colonoscopy was negative for a gastrointestinal primary malignancy. She was taken to the operating room by a gynecologic oncologist who performed robotic assisted total laparoscopic hysterectomy, bilateral salpingo-oophorectomy and sentinel pelvic lymph node dissection. Findings at the time of surgery included omental adhesions to the left fallopian tubes, grossly normal uterus, bilateral adnexa, appendix, and omentum. Final pathology showed a mucin producing invasive adenocarcinoma with focal squamous differentiation which involved the entire endometrial cavity and serosa but spared the cervix. The bilateral adnexa and all lymph nodes were negative for metastases. The pelvic washings were negative. Immunohistochemical studies were compatible with iMACE (Table 2). The signet-ring cell component of the carcinoma was focally weakly to moderately positive for cytokeratins 7 and 20, positive for CDX2, negative for PAX8 and estrogen receptors and focally, very weakly positive for E-cadherin (Fig. 1). In situ hybridization analysis for high-risk HPV subtypes showed rare punctate signals in some tumor cells, the interpretation of which was uncertain. The staining for the DNA MMR proteins was intact. She was referred to radiation oncology for adjuvant therapy, but the patient declined any further treatment. Patient has been seen for regular follow-up with her gynecologic oncologist and is clinically without evidence of disease at 36 months since surgery.

Case C A 73-year-old para 3, compliant with routine well-woman gynecologic care, presented to the emergency department with two months of post-menopausal bleeding. Pelvic ultrasound revealed an endometrial thickness of 6 mm. She had a pertinent gynecologic history of right salpingo-oophorectomy for a tubo-ovarian abscess and cervical dysplasia status post loop electrode excisional procedure in the distant past with subsequent normal Pap smears. A Pap smear was performed which showed atypical cells of undetermined significance, HPV positive (high risk positive, 16/18/45 negative). Patient was evaluated by a gynecologic oncologist where an abnormally hardened cervix was appreciated, and cervical biopsy, endocervical curettage and endometrial biopsy were performed. On rectovaginal exam there was suspicion for left parametrial shortening. Patient was referred to medical oncology for neoadjuvant chemotherapy for presumed advanced cervical or endometrial primary malignancy. Patient declined chemotherapy and was referred to gastroenterology for repeat screening, with upper and lower endoscopy noncontributory. She underwent a radical abdominal hysterectomy, left salpingo-oophorectomy, and pelvic lymphadenectomy. Intra-operative findings included a flush, hardened cervix with a small amount of necrotic tumor prolapsing through the endocervix. The parametria were grossly not involved, the rectosigmoid colon was densely adherent to posterior uterine wall and the appendix and omentum were grossly normal. Immunohistochemistry confirmed intestinal differentiation (Table 2). The signet-ring cell component of the carcinoma was negative for cytokeratin 7 and positive for cytokeratin 20 (suggesting a colorectal and appendiceal-type immunohistochemical profile), positive for CDX2, negative for PAX8 and estrogen receptors, and completely negative for E-cadherin (Fig. 1). MMR staining was not performed, but next generation sequencing showed the tumor to be MSI stable. The patient expired six months after the initial surgery.

## 3 Discussion

Mucinous carcinoma with intestinal differentiation is a rare variant of endometrial

cancer. Recently, Ardighieri reported a total of nine cases of this rare variant arising in patients between 49 and 81 years of age (Table 1) (Ardighieri et al., 2020, Berger, 1984, Trippel et al., 2017, Rubio et al., 2016, Nieuwenhuizen, 2007, Buell-Gutbrod, 2013, Mogor et al., 2019, Zheng et al., 1995). Interestingly, intestinal differentiation can manifest with a wide spectrum of morphologies, including goblet cells and other gastrointestinal-type cells, such as signet ring cells. In addition to the morphologic features, immunohistochemical markers of gastrointestinal differentiation were positive in the majority of the cases reported. These markers include CK20, CK7, CDX2, villin, and MUC6. Conversely, PAX8 and estrogen and progesterone were usually negative, indicating a loss of Mullerian markers (Ardighieri et al., 2020, Berger, 1984, Trippel et al., 2017, Buell-Gutbrod, 2013, Mogor et al., 2019).

**Table 1:** Literature review of intestinal type mucinous adenocarcinoma.

Case	Age, presenting symptom	Surgery	FIGO stage	Adjuvant	MMR	FU
Case A	80, PMB	H + BSO + PLND	IVB	Declined	Intact	DOD @11 months
Case B	63, PMB	H + BSO + PLND	IIIA	Declined	Intact	NED @36 months
Case C	73, PMB	H + LSO + PLND	4A	Declined	MSI stable	DOD@ 6 months after surgery
Ardighieri et al. (2020)	49, PMB, PP	H + BSO + PLND	IVB	NCHT: Carboplatin, Paclitaxel	Loss: MSH2 MSH6, MSI-H	ALD @6 months
Mogor et al. (2019)	58, NR	H + BSO + PLND	IA	EBRT, VBT	NR	Vaginal and vulvar recurrences. NED@ 87 months
Trippel et al. (2017)	62, PMB	H + BSO + PLND	IA	NRc	Loss: MLH1 PMS2, MSI-H	Peritoneal recurrence. DOD @21 months
Rubio et al. (2016)	81, PMB	H + BSO	IIIA	Declined	NR	NR
Buell-Gutbrod (2013)	55, PMB	H + BSO	IA	NRc	NR	NR
Nieuwenhuizen (2007)	NR	NR	NR	NR	NR	NR
Nieuwenhuizen (2007)	NR	NR	NR	NR	NR	NR
Zheng et al. (1995)	71, PMB	H + BSO + PLND + PALD + OB + AP	IIIC	PRT	NR	Peritoneal recurrence AWD @14 months
Berger (1984)	72, PMB	H + BSO	IA	NR	IA	NED

FU: follow up; LUS: lower uterine segment; PMB: post-menopausal bleeding; DOD: Dead of disease; NR: not reported; NRc: Not recommended; H: hysterectomy; BSO: bilateral salpingo-oophorectomy PLND: pelvic lymph node dissection; PALD: para-aortic lymphadenectomy; OB: omental biopsy; AP: appendectomy; MHI-H: high-grade microsatellite instability; NCHT: neoadjuvant, chemotherapy; PRT: pelvic radiation therapy; EBRT: external bean radiation therapy; VBT: vaginal brachytherapy; NED: No evidence of disease.

**Table 2:** Summary of Immunohistochemical Results.

IHC	Case A	Case B	Case C
CK20	+	+	Focally weak
CDX2	+	Focally positive	+
CK7	+	–	–
PAX-8	Focally weak	–	–
ER	–	–	–
E-Cadherin	Focally weakly positive	Focally weakly positive	–

Intestinal differentiation has been postulated by Ardighieri et al. to denote a more aggressive variant of mucinous adenocarcinoma of the endometrium. The stage of disease at presentation was IA for 50% (4/8) of the cases described. However, of the patients with low-stage disease and a follow-up available, two (2/3) had disease recurrence in the vagina, vulva, and peritoneum (Ardighieri et al., 2020). This demonstrated that, despite early stage of presentation, the disease often recurs. All three cases presented with late stage disease and two patients died of disease, with the third disease free at three years post therapy. (Table 2). This not only further confirms Ardighieri’s findings, but also indicates that those individuals with signet-ring cell morphology may have a potentially worse prognosis.

E-cadherin is a calcium-dependent cell-to-cell adhesion molecule found in epithelial tissue and implicated in cellular migration. Alterations in E-cadherin expression have been linked to decreased cell–cell adhesion, increased metastatic potential, tumor dedifferentiation, and deep myometrial invasion in endometrial carcinoma. Wang et al. showed that the signet ring cell component of colorectal adenocarcinomas was often negative for E-cadherin, and the loss of E-cadherin expression conferred a worse clinical prognosis (Wang et al., 2016). In their turn, Humar et al. showed that the loss of E-cadherin initiated carcinogenesis of gastric signet ring cell carcinoma (Humar et al., 2009). Consistent with the previous studies, our series showed that the expression of E-cadherin was lost or markedly decreased in the signet ring cell component of endometrial mucinous adenocarcinoma, although the low number of extant cases does not allow to determine its influence on the clinical behavior of these tumors.

The presence of signet ring cells and other morphologic features of intestinal differentiation in uterine tumors increases the probability of theses tumors being metastases from extragenital organs. Thus, ruling out metastasis from the gastrointestinal system or the breast is imperative. Immunohistochemistry has been shown to have limited value, since both gastrointestinal metastases and intestinal-like mucinous endometrial carcinomas express intestinal-like markers and are negative for PAX8 and estrogen and progesterone receptors. Thus, to rule out metastasis, the time-consuming process of imaging and endoscopy must be performed (Mogor et al., 2019). The differential diagnosis of the cases presented also includes intestinal differentiation in mucinous adenocarcinoma of other regions of the female genital tract, including the cervix and ovary. This can be particularly challenging in advanced-stage disease, when involvement of multiple regions of the female genital tract can be seen at time of diagnosis. It has been proposed that negative HPV molecular testing, negative immunostaining for p16, or both, can aid in excluding an HPV-related primary cervical carcinoma (Trippel et al., 2017). However, the relationship between HPV and the intestinal phenotype is not well understood. An argument can be made for designating case C of our series as a primary cervical carcinoma, given the extensive involvement of the cervix. However, p16 immunostain was negative, which favors either an endometrial origin, or a rare gastric-type adenocarcinoma of the cervix, which is not related to HPV. The latter two diseases would be difficult to differentiate from each other, especially if the tumor centers on the LUS and involves both the cervix and the corpus uteri.

Traditionally, the treatment and prognosis of endometrial carcinoma have relied on histotype and grade of the tumor. This system has been challenged by the development of the ProMise (Proactive Molecular Risk Classifier from for Endometrial Cancer) algorithm which classifies endometrioid adenocarcinoma into four distinct prognostic subtypes and is based upon genomic abnormalities. MSI-H is one such subtype and is classified as a tumor with MLH-1- promoter hypermethylation or mutations of the DNA MMR genes. Recently, Ardighieri and Trippel postulated that this tumor classification system can potentially be extended to iMACE due to the loss of MSH2/MSH6 and the presence of MLH-1- promoter hypermethylation, respectively (Ardighieri et al., 2020, Trippel et al., 2017). These genomic abnormalities were not seen in our cases.

In summary, we describe three rare cases of intestinal type mucinous adenocarcinoma with signet-rings cells. These cases demonstrate the aggressive nature of intestinal differentiation in the endometrium and suggests that the presence of signet-rings cells and loss of E-cadherin expression may render a poorer prognosis as seen in other parts of the body. However, more cases are needed to further study the biologic behavior of this morphological variant. This case report also adds to the body of literature that supports the concept of divergent molecular profiles, including cell adhesion molecule expression, in different types of endometrial carcinoma.

## CRediT authorship contribution statement

**Kieran Seay:** Visualization. **Bethany Bustamante:** Conceptualization, Writing – review & editing. **Alexander Truskinovsky:** Resources, Writing – review & editing. **Andrew Menzin:** Resources. **Gary L. Goldberg:** Project administration, Writing – review & editing, Supervision.

## Declaration of Competing Interest

The authors declare that they have no known competing financial interests or personal relationships that could have appeared to influence the work reported in this paper.
